# The Harder You Work, the Higher Your Satisfaction With Life? The Influence of Police Work Engagement on Life Satisfaction: A Moderated Mediation Model

**DOI:** 10.3389/fpsyg.2019.00826

**Published:** 2019-04-10

**Authors:** Ting Liu, Xiaoqing Zeng, Meirong Chen, Ting Lan

**Affiliations:** ^1^School of Psychology, Jiangxi Normal University, Nanchang, China; ^2^Department of Education, Nanchang Normal University, Nanchang, China

**Keywords:** police officer, work engagement, work-family conflict, psychological detachment, life satisfaction

## Abstract

**Background:** Life satisfaction is a key component of quality of life; it is associated with many factors, including occupational and family life. The results of existing studies examining associations among work engagement, work-family conflict, and life satisfaction have been inconsistent.

**Objective:** We explored the mechanism of action of police work engagement on life satisfaction, and analyzed the relationships among work engagement, work-family conflict, psychological detachment, and police life satisfaction from the angle of family and work relationships.

**Methods:** A total of 760 police officers completed the Utrecht Work Engagement Scale, Satisfaction with Life Scale, Work-Family Conflict Scale, and Psychological Detachment Scale; 714 questionnaires were valid.

**Results:** Work engagement both directly affected police life satisfaction (β = 0.58, *p* < 0.001), and indirectly influenced police life satisfaction through work-family conflict (β = -0.07, *p* < 0.05). Different levels of psychological detachment moderated both the relationship between work engagement and work-family conflict (β = 0.17, *p* < 0.001), and the relationship between work-family conflict and life satisfaction (β = 0.07, *p* < 0.05).

**Conclusion:** A moderated mediation model was established. Work-family conflict partially mediates the relationship between work engagement and police life satisfaction. Psychological detachment moderates the first and second half of the mediating process by which work engagement affects police life satisfaction through work-family conflict.

## Introduction

Life satisfaction comprises individuals’ overall cognitive evaluation of their living conditions for most of the time or for a certain period of time according to criteria of their choice and is a key parameter in the quality of life measure (Shin and Johnson, 1978). As an important positive psychological quality, life satisfaction is also a key component of a subjective sense of happiness. Individuals may have positive or negative emotional experiences based on their cognitive evaluations of their quality of life (Diener, 1984). Specifically, when individuals’ evaluations indicate higher life satisfaction, they experience more positive emotions and fewer negative emotions, and their sense of happiness increases accordingly. As one of the key components of the research field of positive psychology, life satisfaction has drawn special attention from many researchers. Several studies have shown that life satisfaction is closely related to occupation, education, income, leisure, family (Daraei and Mohajery, 2013; Loewe et al., 2013; Kuykendall et al., 2015), and physical and mental health (Gana et al., 2013; Friedman and Kern, 2014; Choi et al., 2017). It may be inferred that life satisfaction has a critical influence on an individual’s social life and psychological behavior.

Little is known about the unique psychological factors that affect the police in China. Specifically, Chinese police have a heavy workload and strict working time regulations. They often work under stressful conditions, and it is common for them to work overtime to deal with emergencies, which, in turn, places increased psychological pressure on them, leading to physical and mental exhaustion. Furthermore, policing is a risky service with a high rate of casualties. Data from the National Bureau of Statistics of China (2016) suggests that the average life expectancy in China had reached 76.34 years, but the average life expectancy of Chinese police officers is only 48 years. As public servants who serve the masses, ensure the safety of people’s lives and property, and maintain social order, police officers bear the responsibility for maintaining national security and social stability. Their happiness, satisfaction with life, and mental state are factors that are not only related to their quality of life but also to the success or failure of governing the country. Improving the mental health and life satisfaction of police officers serves as the foundation for deepening the reform of the police force and promoting social harmony. Life satisfaction among police officers should be improved to help them operate more effectively and protect the citizens. Therefore, it is of great practical significance to explore the life satisfaction of police officers.

Among the factors affecting life satisfaction, work is an important one. The work requirements of the police are unique in that the profession requires a high level of engagement with work. Work engagement is a positive, full, persistent, and diffuse emotional and cognitive state related to work, and includes three dimensions—vigor, dedication, and absorption (Schaufeli et al., 2002). This definition implies that work engagement is based on pleasure and activation which are two dimensions of happiness. Work engagement itself is a positive experience and reflects a high level of vigor, engagement, and a strong sense of identity in work. According to the collective model of job involvement (Rabinowitz and Hall, 1977), work engagement is the outcome of the interaction between personal characteristics and the work situation, and the outcome of work engagement is embodied by employees’ work satisfaction, work performance, and resignation rate, among other factors. After this model was put forward, many studies on work engagement found that it had a positive impact on individuals, was positively related to work satisfaction, negatively correlated with intent to resign, and could predict work satisfaction (Saks, 2006; Alarcon and Edwards, 2011; Airila et al., 2012; Mache et al., 2014). In addition, work engagement was found to be negatively correlated with unhealthy index variables and depressive symptoms (Hakanen and Schaufeli, 2012; Shimazu et al., 2012). Therefore, this study hypothesized that police work engagement would relate positively to life satisfaction. Also, this study further explored the impact of police work engagement on life satisfaction and the underlying mechanism of action.

The relationship between work and family is often inseparable. In addition to work responsibilities, people also have to take care of their families, and the collision of these two roles can lead to conflict. Work-family conflict (Greenhaus and Beutell, 1985) usually refers to a kind of inter-role conflict that occurs when work roles and family roles are incompatible in many ways, leading to either work-family conflict or family work conflict. The two-way model of work-family conflict (Frone et al., 1992) holds that work engagement and work stressors (e.g., work stress, low autonomy at work, role ambiguity) jointly contribute to work-family conflict; family engagement and family stressors (parents’ burden, children’s misbehavior) jointly contribute to family work conflict. Previous studies have shown that work-family conflict generally exists in the police community (Yun et al., 2015), is significantly correlated with life satisfaction (Erdamar and Demirel, 2016; Prajogo, 2016; Yucel, 2017), and may even lead to serious physical and mental illness and adverse conditions. In contrast, physical and mental health are key criteria for life satisfaction (Graham et al., 2007). Related studies show that work-family conflicts are often related to negative states such as stress and burnout. Greater work stress leads to a high degree of job involvement which, in turn, leads to greater perceived work-family conflicts (Eby et al., 2005; Peter et al., 2016). However, the role accumulation hypothesis (Sieber, 1974) posits that an individual’s multiple roles can positively influence one another. The positive spillover (Crouter, 1984) hypothesis, which is based on the role accumulation hypothesis, holds that resources are malleable, and resources such as time and energy can be shared and developed into different fields to interact with each other. Therefore, the resources and benefits individuals obtain by performing the activities associated with one of their roles can positively spill into their other roles, and people can transfer their feelings, attitudes, and behaviors established through their work into the family domain. Existing studies show that work engagement can affect an individual’s positive emotions in the family domain through positive emotion spillover effects (Culbertson et al., 2012) and reduce work-family conflicts. In addition, an individual’s role activities and work engagement are also beneficial to his/her family role (Crouter, 1984; Barnett and Hyde, 2001; Rothbard, 2001). Therefore, police work engagement may affect life satisfaction by reducing work-family conflicts.

Based on previous research, Hypothesis 1 is as follows: work-family conflict plays a mediating role in the impact of work engagement on police life satisfaction.

Individuals consume resources in response to work and family needs. Increasing resource consumption in one area will inevitably lead to the reduction of available resources in another field. When individual resources cannot be restored and replenished, conflicts may arise. Therefore, the recovery of one’s own resources is an effective way to alleviate the working-family conflict and its negative consequences. Studies have shown that psychological detachment from work during non-working hours is an important strategy for this recovery (Sonnentag and Fritz, 2007). Psychological detachment refers to separation from work in terms of both time and space wherein an individual disengages from and stops thinking about work-related issues after work (Sonnentag and Bayer, 2005). With psychological detachment, the individual is both physically and mentally separate themselves from the workplace. Psychological detachment can free people from the tasks that deplete their resources. The conservation of resource theory (Hobfoll, 1989) maintains that a recovery experience can help employees replenish depleted resources because of job demands. Without such recovery, especially after prolonged work periods, the individual’s low resource level will become apparent (e.g., fatigue will increase). Studies have shown that psychological detachment is a protective factor in the stressor-stress response relationship, and such detachment helps an individual recover from resource depletion (Sonnentag et al., 2010b; Park et al., 2011). The workload of individuals with high levels of psychological detachment has less of an impact on their marital satisfaction (Germeys and Gieter, 2016). In contrast, when levels of psychological detachment are low, the correlation between job demands and happiness will be stronger, as will the negative correlation between job demands and work engagement (Sonnentag et al., 2010a). This suggests that psychological detachment has a moderating effect on work-family conflict and on an individual’s happiness (Moreno-Jiménez et al., 2009; Virga and Paveloni, 2015). Therefore, psychological detachment not only alleviates the work-family resource competition and depletion but also adjusts pressure and stress responses. Psychological detachment may help police officers release negative emotions, alleviate work-family conflicts, and improve work-related behaviors, thus improving their life satisfaction.

Based on this background information, Hypothesis 2 is as follows: the mediating role that work-family conflict would play in the relationship between work engagement and police life satisfaction would be moderated by psychological detachment.

**FIGURE 1:**
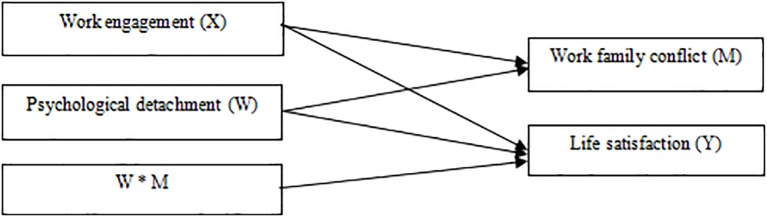
A moderated mediating model.

In sum, the present study constructed a moderated mediation model to test whether work-family conflict mediates the relationship between police work engagement and life satisfaction. Another purpose was to investigate whether psychological detachment moderates this mediating process. The hypothesis model is shown in Figure 1.

## Materials and Methods

### Participants

This study entailed the use of a questionnaire survey. The sample consisted of 760 police officers from all over the country who were undergoing their education and training at a police academy in Jiangxi Province, China, from October to December 2017. All participants signed an informed consent form before completing the questionnaire. We distributed 760 questionnaires, and all were returned. Questionnaires with partially missing data (i.e., partially complete surveys) or duplicate data, potentially from random responding, were excluded, leaving 714 valid questionnaires, which corresponded to an effective response rate of 93.9%. Participants included 549 males and 165 females, whose average age and mean seniority were 32.59 years (*SD* = 9.1) and 10.59 years (*SD* = 9.11), respectively. There were 153 officers from a crime division (21.4%), 156 traffic police (21.8%), 83 household registration officers (11.6%), 27 patrol officers (3.8%), 9 fire fighters (1.3%), and 286 from other categories (40.1%). In terms of education level, there were 9 who had a senior high school diploma or below (1.3%), 144 who had a college diploma (20.2%), 515 who had a bachelor’s degree (72.1%), and 46 who had a master’s degree (6.4%). This study was approved by the Ethics Committee of the School of Psychology of the Jiangxi Normal University.

### Measurements

#### Utrecht Work Engagement Scale

The Chinese version of the fifteen-item Utrecht Work Engagement Scale, developed by Schaufeli et al. (2002) and translated by Yiwen and Yiqun (2005), was used. All items were rated on a 7-point Likert scale ranging from 0 (never) to 6 (always), with higher scores indicating higher work engagement. The scale includes three subscales: “vitality,” “dedication,” and “concentration.” In this study, the Cronbach’s α coefficient of the scale was 0.94, indicating excellent internal consistency.

#### Satisfaction With Life Scale

The Chinese version of the five-item Satisfaction with Life Scale, developed by Diener et al. (1985) and translated by Chunmiao and Xingchang (2009), was used to assess police officers’ life satisfaction. Participants were asked to make a subjective assessment of their overall life satisfaction. A 7-point Likert scale was used to rate the items from 1 (strongly disagree) to 7 (strongly agree), with higher scores indicating higher life satisfaction. In this study, the Cronbach’s α coefficient of the scale was 0.91, indicating excellent internal consistency.

#### Work Family Conflict Scale

Carlson et al. (2000) ten-item Work-Family Conflict Scale is divided into two subscales of work-family conflict and family work conflict. The Chinese version was revised by Hemiao (2007) and adopted and verified by other studies (Surong et al., 2017). Participants rated each item on a 5-point Likert scale (1 = total non-conformity; 5 = total conformity), with higher scores indicating higher work-family conflict. In this study, the scale’s Cronbach’s α coefficient was 0.87, indicating good internal consistency.

#### Psychological Detachment Scale

There are four items in the Psychological Detachment Scale, a component of the Recovery Experience Questionnaire compiled by Sonnentag and Fritz (2007). For this study, the Chinese version of this scale revised by Hongyu et al. (2014) was used, which has been widely used for measuring psychological detachment. It uses a 5-point Likert scale ranging from 1 (totally inconsistent) to 5 (completely consistent), with higher scores indicating higher levels of individual psychological detachment. In this study, the scale’s Cronbach’s α coefficient was 0.85, indicating good internal consistency.

### Data Analysis

The prior procedural control process of test and common variance analysis were applied to the four questionnaires through the Harman’s single-factor test. Using SPSS 22.0 statistical software, the correlations between variables were tested using bivariate Pearson correlations after descriptive statistics had been computed. To be conservative, gender, seniority, police classification, and education level were included as control variables in subsequent analyses to exclude their influences.

Hypotheses 1 and 2 were tested using moderated mediation analyses. The percentile bootstrap method based on deviation correction was applied, and the SPSS macro program PROCESS (written by F. Andrew and edited by Hayes, 2013) was used. By sampling 5,000 bootstrap samples (each sample number was 714), the robust standard errors and bootstrap confidence intervals of parameter estimation were obtained. Then, the mediating association of work-family conflict in the relationship between work engagement and life satisfaction was tested, controlling for gender, seniority, police classification, and education level. Model 4 was used to test Hypotheses 1, and Model 59 was used to test Hypotheses 2. To determine how psychological detachment moderates the relationship between work engagement, work-family conflict, and life satisfaction, a simple slope test was used. The interaction diagram based on psychological detachment was adopted (one standard deviation above the mean and one standard deviation below the mean).

## Results

### Common Method Biases

Results showed that five eigenvalues greater than one were extracted, and the variance explained by the first factor was 32.13%, which was less than the 40% required by the critical standard (Podsakoff et al., 2003). This shows that there was no significant issue of common method bias in the study data.

### Descriptive Statistics and Correlational Analysis

Gender differences were found in levels of work engagement, work-family conflict, psychological detachment, and life satisfaction (*t* = -2.25, *p* < 0.05, *d* = 0.30; *t* = 2.36, *p* < 0.05, *d* = 0.30; *t* = -2.36, *p* < 0.05, *d* = 0.30; *t* = -6.53, *p* < 0.001, *d* = 0.28). Both work engagement and life satisfaction were significantly different by seniority, police classification, and education level. The degree of psychological detachment significantly differed by police type and seniority. In addition, there was a significant difference in police seniority by work-family conflict.

The descriptive statistics and correlation analysis results of each variable with gender, seniority, police classification, and education level controlled, showed that work engagement was significantly positively correlated with life satisfaction and negatively correlated with work-family conflict. Work-family conflict was positively correlated with psychological detachment and negatively correlated with life satisfaction. There was a significant positive correlation between psychological detachment and life satisfaction, as shown in Table 1.

**Table 1:** Descriptive statistics and correlation matrix of all variables.

	*M*	*SD*	1	2	3	4
1. Work engagement	53.78	14.606	1			
2. Work-family conflict	28.38	7.296	–0.089^∗^	1		
3. Psychological detachment	11.49	3.843	0.069	0.152^∗∗∗^	1	
4. Life satisfaction	22.92	6.464	0.582^∗∗∗^	–0.126^∗∗^	0.192^∗∗∗^	1


*N = 714; ^∗^*P* < 0.05, ^∗∗^*P* < 0.01, ^∗∗∗^*P* < 0.001.*

### Moderated Mediation Analyses

Correlation analysis showed that the relationship between work engagement, work-family conflict, and life satisfaction met the conditions for the mediating effect test.

**FIGURE 2:**
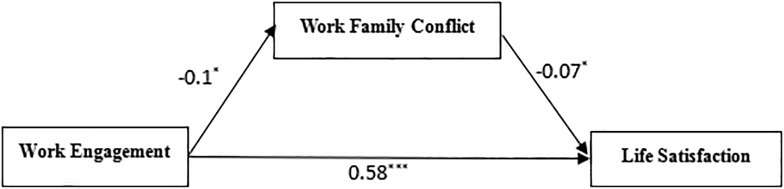
Path coefficients of work engagement, work-family conflict and life satisfaction. ^∗^*P* 0.05, ^∗∗∗^*P* 0.001.

The PROCESS macro for SPSS (Model 4) was used to test Hypothesis 1. Table 2 shows that work engagement had a significant negative association with work-family conflict (*t* = -2.39, *p*< 0.05). When both work engagement and work-family conflict were entered into the regression equation, work engagement had a significant positive association with life satisfaction, and work-family conflict had a significant negative association with life satisfaction, with the bootstrapped confidence intervals of the mediating association not containing zero [(-0.13, -0.014)]. Therefore, work-family conflict plays a partial mediating role in the relationship between work engagement and life satisfaction. Thus, Hypothesis 1 was supported. The detailed path model is shown in Figure 2.

**Table 2:** Regression analysis results of the mediating role of work-family conflict between work engagement and life satisfaction.

Regression Equation		Overall model fit			Significance of regression coefficient			
Outcome	Predictor	*R*	*R^2^*	*F*	β	LLCI	ULCI	*t*
Work-family conflict	Gender	0.15	0.02	3.07**	–0.20	–0.37	–0.02	–2.22*
	Police classification				0.027	–0.01	0.06	1.44
	Seniority				–0.003	–0.01	0.006	–0.56
	Education level				0.025	–0.14	0.16	–0.36
	Work engagement				–0.1	–0.17	–0.02	–2.39*

Life satisfaction	Gender	0.65	0.42	86.00***	0.39	0.25	0.52	5.61***
	Police classification				–0.04	–0.07	–0.01	–2.77**
	Seniority				0.001	–0.006	0.008	0.36
	Education level				0.02	–0.09	0.13	0.38
	Work engagement				0.58	0.52	0.64	18.8***
	Work-family conflict				–0.07	–0.13	–0.014	–2.45*


*All the variables in the model were normalized and then substituted into the regression equation. LLCI, lower level confidence interval. ULCI, upper level confidence interval. ^∗^*P* < 0.05, ^∗∗^*P* < 0.01, ^∗∗∗^*P* < 0.001*.

The PROCESS macro for SPSS (Model 59) was used to test Hypothesis 2. In the direct path, the interaction terms of work engagement and psychological detachment had no significant association with life satisfaction (β = 0.04, *t* = 1.85, *p* > 0.5), indicating that psychological detachment did not moderate the direct path. In the first half of the path, work engagement had a significant negative association with work-family conflict (β = -0.13, *t* = -3.41, *p <* 0.001), psychological detachment had a significant positive association with work-family conflict (β = 0.16, *t* = 4.51, *p <* 0.001), and the interaction items of work engagement and psychological detachment had a significant positive association with work-family conflict (β = 0.17, *t* = 5.74, *p <* 0.001). This suggests that psychological detachment plays a moderating role in the relationship between work engagement and work-family conflict. In the second half path, work-family conflict had a significant negative association with life satisfaction (β = -0.11, *t* = -3.72, *p <* 0.001), work engagement had a significant positive association with life satisfaction (β = 0.55, *t* = 17.84, *p <* 0.001), psychological detachment had a significant positive association with life satisfaction (β = 0.15, *t* = 5.36, *p <* 0.001), and the interaction items of work-family conflict and psychological detachment had a significant positive association with work engagement (β = 0.07, *t* = 2.46, *p <* 0.05). This suggests that psychological detachment plays a moderating role in the relationship between work-family conflict and life satisfaction, thus, Hypothesis 2 was supported. See Table 3.

**Table 3:** Regression analysis results of psychological detachment moderate the mediation process.

Regression Equation		Overall model fit			Significance of regression coefficient			
Outcome	Predictor	*R*	*R^2^*	*F*	β	LLCI	ULCI	*t*
Work-family conflict	Gender	0.30	0.09	9.80^∗∗∗^	–0.21	–0.38	–0.04	–2.45*
	Police classification				0.024	–0.01	0.06	1.33
	Seniority				–0.003	–0.01	0.005	0.75
	Education level				–0.008	–0.14	0.13	–0.12
	Work engagement				–0.13	–0.21	–0.06	–3.41***
	Psychological detachment				0.16	0.09	0.23	4.51***
	Work engagement×Psychological detachment				0.17	0.11	0.23	5.74***

Life satisfaction	Gender	0.67	0.45	72.4***	0.34	0.21	0.47	5.02***
	Police classification				–0.04	–0.07	–0.01	–2.57*
	Seniority				0.0004	–0.006	0.007	0.12
	Education level				0.013	–0.09	0.12	0.23
	Work-family conflict				–0.11	–0.17	–0.05	–3.72***
	Work engagement				0.55	0.49	0.61	17.84***
	Psychological detachment				0.15	0.10	0.21	5.36***
	Work engagement×Psychological detachment				0.04	–0.003	0.09	1.85
	Work-family conflict×Psychological detachment				0.07	0.014	0.12	2.46*


*All the variables in the model were normalized and then substituted into the regression equation. LLCI, lower level confidence interval. ULCI, upper level confidence interval. ^∗^*P* < 0.05, ^∗∗^*P* < 0.01, ^∗∗∗^*P* < 0.001.*

**FIGURE 3:**
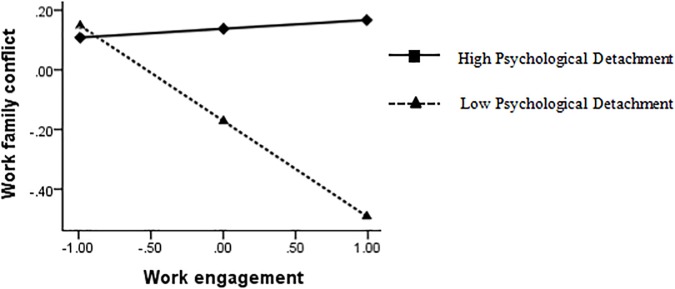
Simple slopes of psychological detachment moderate the relationship between work engagement and work-family conflict.

**FIGURE 4:**
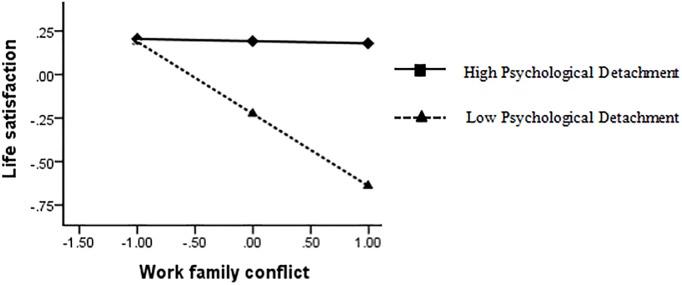
Simple slopes of psychological detachment moderate the relationship between work-family conflict and life satisfaction.

The interaction diagram directly reflects how the influence of work engagement on work-family conflict and life satisfaction was moderated by psychological detachment (see Figure 3, 4). When the degree of work engagement was higher, the participants with high psychological detachment showed higher work-family conflict, while the participants with low psychological detachment showed lower work-family conflict. When the degree of work-family conflict was higher, officers with high psychological detachment showed higher life satisfaction, whereas those with low psychological detachment showed lower life satisfaction.

In sum, the moderated mediation model was established (Hayes, 2012). Work-family conflict mediated the relationship between work engagement and police life satisfaction, and at the same time, psychological detachment moderated the mediating process by which work engagement potentially affects police life satisfaction through work-family conflict.

## Discussion

The occupational demands of police work are high. This job requires officers to fight against illegal and even violent acts that endanger people’s lives and property. Police work also demands that officers be dedicated to perceiving and preventing illegal incidents at any time. Thus, police work requires high levels of work engagement. The outcome of the present study shows that work engagement is positively related to police life satisfaction, which is consistent with previous findings (Hakanen and Schaufeli, 2012). On this basis, we introduced work-family conflict and psychological detachment to further explore the mechanism by which work engagement affects police officers’ life satisfaction and to explain how work engagement affects their life satisfaction from the perspective of family and work relationships.

### The Mediating Role of Work-Family Conflict

The outcome of the present study shows that work engagement has a significant negative correlation with work-family conflict, which, in turn, has a significant negative correlation with police life satisfaction. This verified the study hypothesis that work-family conflict plays a part in mediating the relationship between work engagement and police life satisfaction. Therefore, work-family conflict is an important factor in determining the influence of work engagement on police officers’ life satisfaction. This suggests that police officers’ life satisfaction can be improved by reducing work-family conflicts from the perspective of work-family relationships.

Many studies have found that the higher the work commitment, the higher the work-family conflict (Carlson et al., 2000; Byron, 2005). The results of the present study seem to be inconsistent with many previous studies, which may be due to a number of factors. Firstly, Bennett and Bell (2004) proposed in the high job engagement model that work characteristics cannot only directly affect employees’ work engagement but also indirectly through intrinsic motivation. The internal psychological states such as internal drive, satisfaction, demand, motivation and so on provided by the work characteristics, are the most important factors that drive employees’ personal behavior. Some people’s motivation for work comes from the interest or curiosity from the job itself (which is intrinsic motivation, or the quality or attraction derived from the job). In China, there is a high value placed on serving the country, and individuals in the police profession may therefore feel a sense of honor in their jobs. As a result, the professional identity and commitment of the police are high, and their work commitment is mostly driven by their internal motivation. The mood-as-input model (Martin et al., 1993), which explains the intrinsic motivation of work engagement, suggests that when people use their own cognitive criteria to evaluate tasks that have no clear results, they tend to use mood-based information as a basis for stopping or continuing tasks; this judgement approach is known as the “enough stop rule.” Specifically, the “enough stop rule” is a frame of reference for a person to decide whether to reach the goal or whether to stop trying. When people think that the goal has been achieved, a positive state of mind is generated. Intrinsic motivation is a motivational antecedent of work engagement, with the latter aimed at achieving job-related pleasure and contentment. The police participants in this study are responsible for maintaining public order and ensuring the safety of people’s lives. When they find that because of their hard work and engagement the community under their jurisdiction is in good order and that no incident affecting the personal and property security of the residents has occurred, the police feel gratified and satisfied. This positive state of mind enables participants to apply the “enough stop rule” to readjust time and energy distribution between work and family, thereby reducing work-family conflicts.

Secondly, Chinese employees are deeply influenced by Chinese traditional culture and have a strong sense of work priority. Collectivistic cultures encourage individuals to approach work with dedication and professionalism. Chinese employees generally view work as being a central part of their lives (Mian et al., 2011). Families show understanding and support for family members’ involvement in work given traditional Chinese cultural values. Hard work is not only a means of earning a living but also an expression of responsibility to the country and family. Good performance cannot only bring enough financial security to the family life but also bring honor to the whole family (Mian et al., 2011). Individuals with a high level of work engagement have been found to be accompanied by a strong positive emotion in life (Little et al., 2010), and employees with less negative emotions reported fewer work-family conflicts (Stoeva et al., 2002). Positive emotions associated with work and resources derived from work (e.g., increased self-esteem, financial income) can effectively influence family life, and positive emotional experiences can create a good family atmosphere and strengthen the degree of emotional connection among family members. The income from work engagement can improve the quality of family life and counteract the influence of negative emotions associated with some family factors (e.g., household expenses) on work. At this point, the relationship between work and family is believed to be mutually reinforcing. As a result, some of the adverse effects of work are also accepted by the family, and the conflict between work and family is low.

However, people’s perceptions and views often stem from the deep social structure of a culture and represent shared values, beliefs, norms, or social knowledge (Ford et al., 2007). The theory of social identity suggests that a broad sense of social context and public cognition can shape how people view themselves and their neighbors (Ellemers et al., 2002). From the perspective of identity, the police profession can be viewed as a moral identity internalized by individuals. In China, the whole society, including the family members of the police officers (native families and families of their own), has highly recognized the profession of police officers and is aware of the working conditions of the profession, such as long working hours, high risks, the uncertainty of a work situation, and so on. The police can embody their professional value and sense of honor in their work engagement. They can also gain the understanding and support of their family members while taking on more job responsibilities so that they feel less role of conflict pressure. Therefore, in addition to obtaining recognition and self-esteem through work performance, the individual with high work engagement can also compensate for the resource gap and effectively reduce work-family conflict.

Previous studies have shown that work-family conflict affects an individual’s work and family life (Pattusamy and Jacob, 2016). The outcome of this study is consistent with that of previous studies, with work-family conflict relating negatively with police life satisfaction; thus, the higher the work-family conflict, the lower the individual’s life satisfaction (Kossek and Ozeki, 1998; Willis et al., 2008; Taşdelen-Karçkay and Bakalim, 2017). Firstly, the conflict between work and family leads to a decline in job satisfaction and reduce the performance of employees. Previous studies have shown that conflict is negatively correlated with job satisfaction and a positive correlation with painful emotions (Anderson et al., 2002). Secondly, work-family conflict will reduce an individual’s marriage quality. A study by Mauno and Kinnunen (1999) shows that there is a negative correlation between work-family conflict and marital satisfaction. Finally, a significant correlation was found between work-family conflict and psychological stress and job burnout (Frone et al., 1992). Thus, work-family conflicts are associated with several physical or psychological indicators of diseases related to stress and burnout. When there are organizational values that support balance, employees have a positive emotional state and better physical health, and consequently, their job and life satisfaction are higher (Burke and Nelson, 1998; Gayathri and Karthikeyan, 2016). Similarly, more work-family conflicts among individual police officers can lead to a decline in marital satisfaction, by extension affecting the quality of family life, reducing job satisfaction and performance, and increasing psychological stress. Consequently, this results in job burnout and reduced life satisfaction.

As far as the police profession is concerned, the spirit of dedication and the prevailing consensus in society to “give up family interests for those of the public” has enabled the police to gain wide support and recognition of their professional roles from all members of society. This is beneficial to their family roles, as their life satisfaction is affected to a greater extent by their degree of work engagement. In addition, the positive experiences through work also reduce work-family conflicts. Therefore, high work engagement not only has a direct impact on life satisfaction but also improves life satisfaction by reducing work-family conflicts.

### The Moderating Role of Psychological Detachment

This study found that psychological detachment is a protective factor for police officers’ life satisfaction. When the conflict between work and family is high, individuals with high levels of psychological detachment show high life satisfaction, whereas those with low levels of psychological detachment show low life satisfaction. The Effort-Recovery Model (Meijman and Mulder, 1998) asserts that individuals must expend effort to cope with job demands, but the effort exerted depletes their resources, and they subsequently develop adaptive physiological responses. However, a short rest can help individuals restore their physical and mental systems to the baseline state. Psychological detachment helps the process of resource recovery. Psychological detachment means that individuals no longer need to expend effort to cope with job demands, and thus the functional systems awakened during work rest, and physical and mental resources are replenished (Sonnentag and Bayer, 2005). Psychological detachment has a significant negative correlation with emotional exhaustion and a significant positive correlation with positive emotions and relaxation experiences (Sonnentag et al., 2008). This variable is also significantly correlated with individuals’ job performance (Fritz et al., 2010). In addition, the direct effects of psychological detachment on employees’ happiness—including good short-term emotional states and relatively long-term mental health aspects—have been verified by many studies (Davidson et al., 2010; Sonnentag et al., 2012; Shimazu et al., 2016). Psychological detachment can help individuals shape a more positive mindset. Thus, police officers with higher levels of psychological detachment can return to their family roles after work faster. This can help them escape from work-related stress, forget their troubles, and recover the resources they have lost at work. These factors can improve the positive emotional experiences of the individual, thereby reducing work-family conflict. Therefore, in the case of high work-family conflict, police officers with higher levels of psychological detachment may have higher life satisfaction than those with lower levels of psychological detachment.

Interestingly, our study also found that in police officers with low levels of psychological detachment, work-family conflict levels were lower when work engagement increased. In contrast, in police with a high degree of psychological detachment, as work involvement increased, work-family conflict increased, consistent with the results of previous studies (Moreno-Jiménez et al., 2009). This is probably because the police are involved in a highly loyal profession, which is the most important factor in the collectivism culture and the embodiment of high organizational commitment. Individuals with high organizational commitment can be shown as being loyal to the organization, focused on accomplishing organizational tasks and integrating themselves with organizational goals (Hong, 2016). At the same time, the police profession requires a high degree of career commitment. London (1983) asserts that career commitment is the intensity of motivation to pursue career achievement based on professional identity. In short, their definition of career commitment is professionalism. In China, individuals tend to display intense dedication and commitment to their careers at the expense of their family roles. As we have discussed earlier, the working characteristics and family relationships of the Chinese police are accepted by themselves and their families. According to Sonnentag et al. (2012), work engagement can predict recovery level at the end of a day’s work, with job demands and situational constraints as moderating factors. The moderating role of psychological detachment is dependent on the specific job demands and social culture. Therefore, when the degree of psychological detachment of the police is lower, it means that the higher the work engagement, the more professional or career spirit can be reflected, which will help to alleviate the work-family conflict.

In order to better understand the process of transition from work role to family role, Ashforth et al. (2000) put forward the concepts of separation and integration, and considered that separation and integration occupy opposite ends of a continuum, with one end of the continuum being high levels of separation, indicating low ambiguity and clear boundaries between the different social roles of individuals. A police officer with high levels of psychological detachment is at the high separation end of the continuum. High levels of psychological detachment coexist with high boundaries between work and family. These boundaries mean that it will take longer for the police to successfully return to “work mode,” so when police officers are faced with unexpected security situations, overtime and other work-related tasks, previously scheduled family activities have to be abandoned or postponed until later, leading to higher work-family conflict. In addition, job burnout among police personnel may manifest itself in a high degree of psychological detachment. Maslach et al. (2001) suggested that job burnout is a long-term response to chronic interpersonal stressors at work with three levels, namely overwhelming exhaustion, cynicism, and feelings of ineffectiveness and failure. Increased work engagement among police officers in combination with long-term overload during working hours, eventually produces long-term emotional and physiological exhaustion. In the case of high work-family separation, with the increase of work engagement, psychological detachment will increase the pressure of the role transition and carry the negative emotions caused by job burnout into the family relationship. It will take longer for individuals to return to “work mode” the next morning (De Jonge et al., 2012), which will negatively impact work and lead to higher work-family conflicts. There is no quick transition between the roles of individuals at work and in the family, so work-family conflicts are also on the rise.

### Limitations and Future Research Suggestions

Although our findings provide empirical support for improving police life satisfaction, this study has several limitations.

First, the present study utilized a correlational research design and relied on cross-sectional data. Although these designs are based on theory, they still constrain us from drawing conclusions about causality. To ascertain causality, future studies can involve an intervention group and a control group as well as a longitudinal design.

Second, the majority of participants in this study were male. This gender difference is reflective of the fact that policing is a male-dominated occupation. The gender ratio of participants should be balanced in future studies. Additionally, the present study examined a relatively limited set of outcomes. Gender, seniority, police classification, and education level were included as control variables in the data analysis, but the influence of these variables was not explored. Future research should build on the relevant individual factors affecting police life satisfaction and provide additional empirical support for improvements.

Lastly, this study only investigated Chinese police, which limits the generalizability of our study findings. American employees may have more family needs than Chinese employees, and family needs have a greater impact on work-family conflicts in the United States than in China. In contrast, the level of engagement in work contributes more to the initiation of work-family conflicts in China (Yang et al., 2000). Also, some of our findings were inconsistent with those of previous studies, so whether police forces of different cultural environments demonstrate similar results is worthy of further study. In order to verify the universality of our findings, future research could replicate our model by using cross-national samples to compare police work and family relationships across different cultural groups.

## Conclusion

Based on our analysis and discussion, the current study suggests that work-family conflict mediates the relationship between work engagement and police officers’ life satisfaction. Psychological detachment moderated the first and second halves of the mediation process, in which work engagement affected the police officer’s life satisfaction through work-family conflict.

The results of our study provide both a theoretical and an empirical basis for the development of interventions, and these models show, to some extent, the related variables that affect life satisfaction and the nature of their relationships. According to the results of this study, in terms of improving the life satisfaction of police officers and other employees, we can strengthen the professional identity and commitment of the staff through pre-service training, beginning with the enhancement of internal motivation, thereby increasing the employees’ willingness to engage in their work. In addition, for employees with a high level of enthusiasm for their work, moderate psychological detachment is beneficial for improving their life satisfaction, which provides evidence for the broadening of the research field of positive psychology.

## Ethics Statement

All study participants provided informed consent, and the study design was approved by the appropriate ethics review board.

## Author Contributions

XZ contribute conception and design of the study, TLi performed the statistical analysis, and wrote the first draft of the manuscript. XZ and MC revised it critically for important intellectual content. TLi and TLa collected the raw data and organized the database.

## Conflict of Interest Statement

The authors declare that the research was conducted in the absence of any commercial or financial relationships that could be construed as a potential conflict of interest.
